# Transcranial Magnetic Stimulation (TMS) Plus Modified Constraint-Induced Aphasia Therapy (mCIAT): A Pilot Feasibility Study with Sham and Real cTBS in Two Cases of Chronic Anomia–Protocol Deliverability and Greek Adaptation of mCIAT

**DOI:** 10.3390/brainsci16080780

**Published:** 2026-07-24

**Authors:** Anastasios M. Georgiou, Panagiota Papaioannou, Maria Kambanaros

**Affiliations:** 1 The Cyprus Rehabilitating Aphasia & Dysphagia (C-RAD) Laboratory, Department of Rehabilitation Sciences, Cyprus University of Technology, Limassol 3022, Cyprus; 2 The Brain and Neurorehabilitation Lab, Department of Rehabilitation Sciences, Cyprus University of Technology, Limassol 3041, Cyprus; 3 College of Health, Adelaide University, Adelaide, SA 5000, Australia

**Keywords:** stroke, aphasia, cTBS, intensive aphasia therapy, standalone treatment

## Abstract

**Background/Objectives:** Stroke-related aphasia significantly impairs communication and quality of life (QoL). Constraint-induced aphasia therapy (CIAT) and repetitive transcranial magnetic stimulation (rTMS) are evidence-based approaches targeting language recovery. No combined protocol exists for Greek, a morphologically complex language. The present study was designed as a feasibility pilot, with the primary objective of determining if a modified CIAT (mCIAT) protocol, with or without continuous theta burst stimulation (cTBS), could be delivered safely and acceptably in a Cypriot clinical context, while also reporting the first mCIAT protocol adapted for Greek. **Methods:** A single-subject experimental design (SSED) was employed. Two participants (PAs) with chronic anomic aphasia underwent a 10-day treatment protocol. PA1 received sham cTBS plus mCIAT-GR; PA2 received real cTBS (to the right inferior frontal gyrus) plus mCIAT. Multiple language, cognitive, and QoL measures were administered across three baselines and two follow-up time points. **Results:** Both PAs completed the full protocol with 100% session adherence and no adverse effects, establishing the feasibility of delivery. Neither participant showed improvements in standardized expressive language measures following mCIAT with or without cTBS; Standard Error of Measurement (SEM) analysis confirmed that observed score fluctuations on most measures fell within expected measurement noise. PA2 showed improvement in QoL (SAQOL-39g) domains at the two-year follow-up, though this finding requires cautious interpretation given the follow-up interval and regression-to-mean considerations. **Conclusions:** The Greek mCIAT protocol is feasible and safe to deliver in a Cypriot clinical setting. The absence of clear language improvements may reflect several factors, including ceiling effects, limited assessment sensitivity, insufficient treatment dosage, or limited intervention effects. These findings should be interpreted cautiously and require confirmation in larger, adequately powered studies.

## 1. Introduction

Stroke is a leading cause of aphasia, a communication disability, and aphasia significantly impacts quality of life (QoL) for both the stroke survivor and the family carer. Aphasia can affect the understanding of language, speaking abilities, reading, and writing skills, and carrying out basic arithmetic operations [1]. Chronic aphasia, that is, aphasia greater than 6 months post-stroke, can result in social isolation, mood disorders, depression, and cognitive impairments [2]. Considering the significant impact of aphasia holistically on the overall general health and well-being of the individual living with aphasia and his/her family, the need for effective aphasia therapies becomes crucial. Moreover, accurate information on the aphasic syndrome or aphasia type (e.g., anomic aphasia) and severity level is of paramount importance because these factors are associated with different prognoses. For example, anomic aphasia characterized by intact language comprehension and meaningful speech content but circumlocutions (due to word finding difficulties) is usually the end phase of recovery from other, mild to moderate, aphasia types including Broca’s aphasia [3]. Understanding such prognostic nuances allows clinicians to tailor therapeutic protocols to the specific type of aphasia and its likely progression, optimizing rehabilitation outcomes for post-stroke patients and giving hope to people with aphasia and their families. While prognostic factors in aphasia have been studied extensively (see [4,5]), their integration into combined neuromodulation–behavioural therapy protocols for under-resourced language contexts—such as Greek-speaking Cypriots—remains underexplored [6].

Traditional interventions for aphasia post-stroke include several behavioural strategies (see [7] for a review). One such strategy is constraint-induced aphasia therapy (CIAT), an intensive therapy model based on the forced use of verbal oral language and constraint of compensatory (nonverbal) communication strategies, massed treatment practice over a brief period (usually 3 to 4 h per day for 10 consecutive days) and shaping (inducing) verbal output into more complex responses according to the needs of the individual [8]. The principal method is a basic card game (go-fish type game design) played by 2–3 patients and a speech-language therapist (SLT), where each player is given around 5 cards with pictures of different objects and the goal is to collect as many pairs of matching cards as possible by asking others if they have the exact other card for the pair. Only spoken verbal production is allowed (constraint technique), and screens are sometimes put up between players to prevent them from communicating using other forms (e.g., gesture or writing) and viewing each other’s cards. When treatment begins, all approximately relevant answers are acceptable; however, the shaping technique is gradually introduced to advance the accuracy of the responses, and the SLT can provide additional cueing to promote a successful turn or correct utterance. One limitation of the original stimuli (created in German) is the over-reliance on objects/nouns and the challenge of integrating action words/verbs and function words into the card game [9]. Currently, CIAT programmes are available in very few languages.

Over time, researchers have modified the basic design of CIAT (mCIAT) first, to address implementation challenges in clinical settings including reducing the length (i.e., hours per day) of the treatment sessions [10] and involving communication partners in the treatment [11], second, to explore the use of additional multimodal cues (see [12]) and third, to determine treatment benefits by improving functional communication [13,14]. One major variation to the original CIAT protocol is CIAT II [15] for which a broader range of language activities common to conventional aphasia therapy are incorporated in the treatment sessions including picture description, role-playing activities, and repetition exercises. A second major variation is CIAT Plus [13,14] for which transfer of newly acquired language skills into everyday life is a priority and activities are provided for the individual to practice with at home. CIAT Plus also allows for the use of different stimuli/cards, including photographs of objects and trains individuals to use written language [13]. CIAT is also known by other names including intensive action therapy (IALT) [16,17], and constraint-induced language therapy (CILT) [18]. The current study adapted CIAT for use with Greek-speaking individuals, a morphologically complex language that presents distinct challenges for protocol adaptation. The current study adapted CIAT for use with Greek-speaking individuals, as Greek is a morphologically complex language that presents distinct challenges for protocol adaptation. Standard Greek features rich inflectional morphology—including noun case, verb tense, aspect, and voice distinctions—and the bilectal situation in Cyprus, where speakers use both Standard Modern Greek and Cypriot Greek, adds further complexity to the linguistic demands of treatment stimuli [19]. Verb-argument complexity in Greek, including the morphological marking of passive voice, makes direct adaptation of English- or German-derived CIAT stimuli non-trivial. Moreover, published aphasia treatment research in Greek is sparse (see [7] for review), and no validated Greek mCIAT protocol existed prior to the present study. This underlines the need for language-specific adaptation rather than direct translation, and is the primary justification for reporting the mCIAT adapted for Greek (mCIAT-GR) protocol as a co-equal contribution of the study.

In further efforts to advance aphasia treatments, researchers have also explored noninvasive neuromodulation techniques. One such method involves direct cortical stimulation using repetitive transcranial magnetic stimulation (rTMS). TMS uses electromagnetic induction to generate brief, intense magnetic pulses that penetrate the skull and induce electrical currents in targeted brain regions. These currents can either activate or inhibit neurons, directly influencing brain function in specific areas. TMS can be administered as a single pulse or in a series of pulses, known as repetitive TMS (rTMS). While a single pulse has a very brief effect lasting around 100 ms, rTMS produces effects that extend beyond the stimulation period [20]. The impact of rTMS depends on the pulse frequency: low-frequency (LF) rTMS, usually at 1 Hz, suppresses underlying brain activity, whereas high-frequency (HF) rTMS, typically above 5 Hz, enhances cortical excitability. Research on the benefits of TMS on improving aphasia recovery has investigated its effectiveness at different recovery stages, including post-acute stroke (e.g., [21,22]), subacute stroke (e.g., [23,24]), and chronic stroke (e.g., [25,26,27,28]). While most studies have focused on the effects of LF rTMS applied to the contralesional inferior frontal gyrus (IFG) in conjunction with speech and language therapy (SALT), promising results have also been observed with HF rTMS targeting perilesional tissue in the left frontal regions (e.g., [25,29,30,31]). The foundational evidence for inhibitory rTMS applied to the contralesional IFG in aphasia was established by Naeser and colleagues [32], whose early single-case and small-group studies demonstrated that 1 Hz rTMS to the right pars triangularis (pTr) could produce lasting improvements in picture naming in chronic non-fluent aphasia, and whose 2012 systematic review consolidated the evidence base for this approach across multiple TMS paradigms and aphasia types [33]. These works provide the direct empirical background for the continuous Theta Burst Stimulation (cTBS)-to-right-IFG protocol used in the present study. Compared with conventional low-frequency rTMS protocols, cTBS provides a time-efficient inhibitory stimulation approach, delivering a sustained modulatory effect through a brief stimulation train. This characteristic makes cTBS particularly suitable for integration within intensive behavioural language therapies, where treatment time and patient burden are important practical considerations.

Overall, very few studies in the aphasiology literature have investigated the combination of rTMS and CIAT for language recovery in chronic aphasia post-stroke. Studies that have explored this aphasia treatment combination, report mixed outcomes, as summarized in Table 1. The present study was designed as a feasibility pilot, with the primary objective of determining whether a combined cTBS-plus-mCIAT protocol could be delivered safely and acceptably in a Cypriot clinical context, while also reporting the first mCIAT protocol adapted for Greek.

## 2. Methods

Ethical approval for this study was obtained from the Cyprus National Bioethics Committee (CNBC) (EEBK/EΠ/2017/37). The research was conducted in compliance with the Helsinki Declaration’s principles and the Medical Research Involving Human Subjects Act (WMO). Participants also provided informed signed consent. Moreover, the Case Report (CARE) guidelines set forth by Gagnier et al. [35] for clinical case reporting, were closely adhered to for case study documentation.

### 2.1. Participants

**Figure 1:**
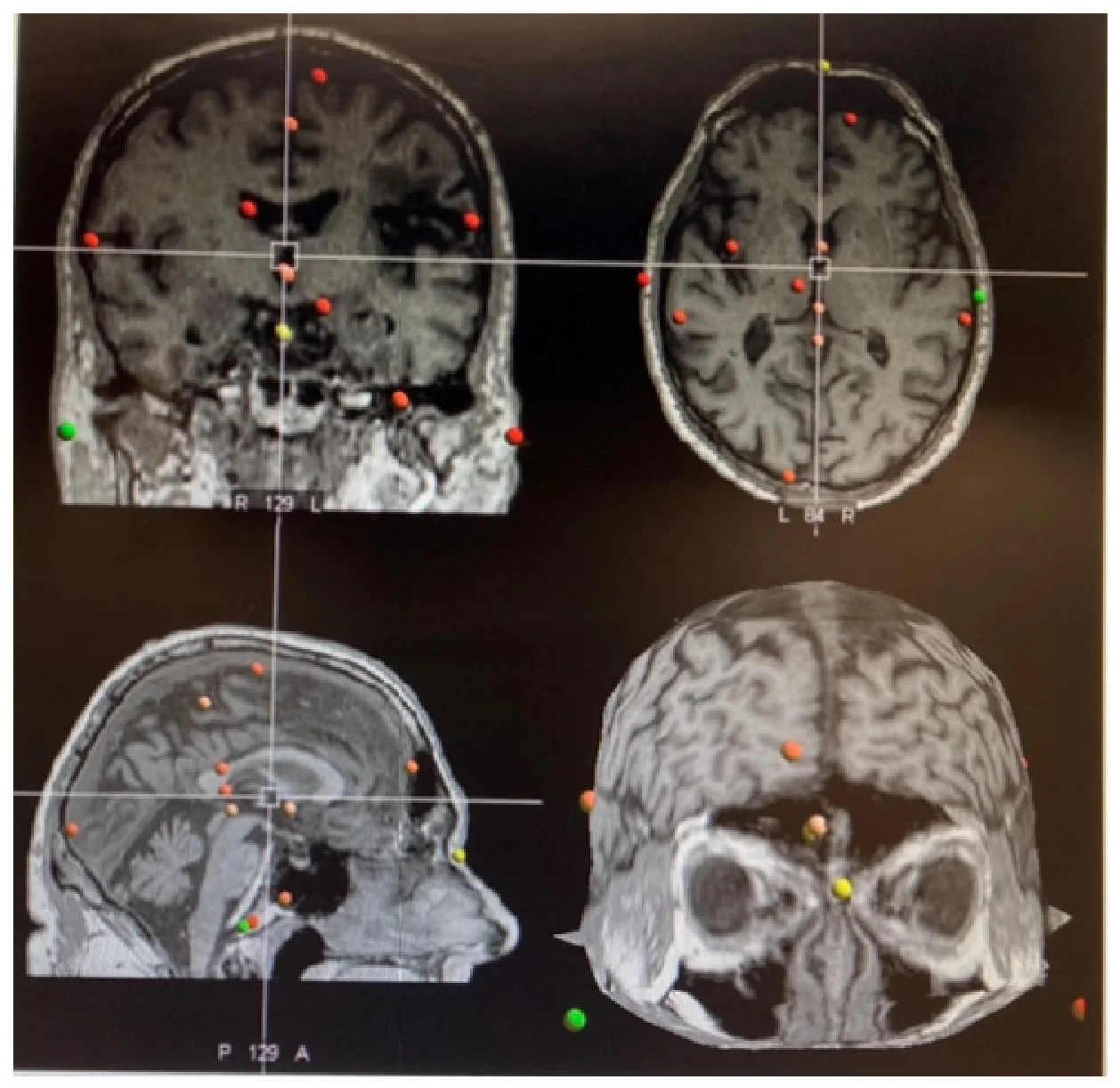
Brain MRI of PA2 six weeks before therapy.

A single-subject experimental design (SSED) trial was undertaken at the University Rehabilitation Clinic of the Department of Rehabilitation Sciences at the Cyprus University of Technology (CUT). Adults who had suffered a single left hemisphere stroke and were in the chronic stage (i.e., at least six months post-stroke) were eligible for recruitment. The inclusion criteria were as follows: (1) aged between 18 and 75 years of age, (2) native speakers of (Cypriot) Greek, (3) right-handed, (4) a diagnosis of a first ever left-sided stroke verified by magnetic resonance imaging (MRI) or computerized tomography (CT), (5) chronic aphasia stage (>6 months poststroke), (6) no history of dementia or other neurological illnesses, and (7) no current participation in any type of language rehabilitation. Exclusion criteria included the following: (1) bilingualism/multilingualism; (2) left-handedness; (3) prior stroke(s); (4) MRI and TMS exclusion criteria; (5) severe dysarthria affecting intelligibility; (6) any other neurological condition affecting the sensorimotor system (e.g., brain tumour); (7) medication that alerts brain excitability to avoid pharmacological influences on TMS; (8) cognitive disorders known before the stroke; and (9) involvement in behavioural language rehabilitation. Two participants (PA1 and PA2) were recruited to the study (see Table 2 for demographic and clinical characteristics). Brain MRI data were available only for PA2 due to the unavailability of PA1’s relevant imaging records; therefore, only PA2’s MRI is presented in Figure 1.

### 2.2. Assessment Tools

Given the pilot nature of the study, a specific primary outcome measure was not selected. Instead, a suite of assessment tools was administered to broadly evaluate language comprehension and production, and to identify measures most responsive to reporting change post therapy. All assessment tools were administered at three baseline measurement times and during follow-up assessments (two times). Regarding the assessment battery, all tools selected are validated in Greek, which constrained the available options. The assessment tools included:

- The Albert’s Test [36] for visual neglect;
- The Raven’s Coloured Progressive Matrices (RCPM) [37];
- The Boston Diagnostic Aphasia Examination–short form (BDAE-SF) [38] excluding the written subtests;
- The short version of the Greek Peabody Picture Vocabulary Test–Revised (PPVT-R) [39] for receptive language;
- The Relative Clauses Task [40];
- The Cypriot-Greek non-word repetition task [19].

QoL was evaluated using the generic form of the Greek version of the Stroke and Aphasia Quality of Life scale-39 item (SAQOL-39g) [41]. The rationale for each measure is as follows. The BDAE-SF was selected as it is the only standardized, validated comprehensive aphasia battery available in Greek; it was administered excluding written subtests to reduce burden. The PPVT-R was included as a measure of single-word receptive vocabulary, where improvement was expected if mCIAT’s receptive card-matching component engaged lexical-semantic access. The Relative Clauses Task [40] provided a morphosyntactically sensitive comprehension measure suited to Greek, targeting a structure known to be vulnerable in agrammatic and anomic aphasia; improvement was hypothesized if treatment generalized to complex syntactic processing. The non-word repetition task [19] was included as a phonological processing measure; given that mCIAT involves a cueing hierarchy that includes phonological cues, improvement in non-word repetition was considered plausible as a secondary phonological outcome. The RCPM was administered as a non-linguistic cognitive control variable: if RCPM scores remained stable while linguistic measures changed, this would suggest language-specific rather than domain-general effects of the intervention, consistent with prior work [26]. The SAQOL-39g was included because functional communication and well-being may be more sensitive to the communicative demands of mCIAT—which requires real conversational exchange—than standardized language tests, and because patient-reported outcomes are increasingly recognized as essential in aphasia rehabilitation research [6].

### 2.3. Procedures

**Figure 2:**
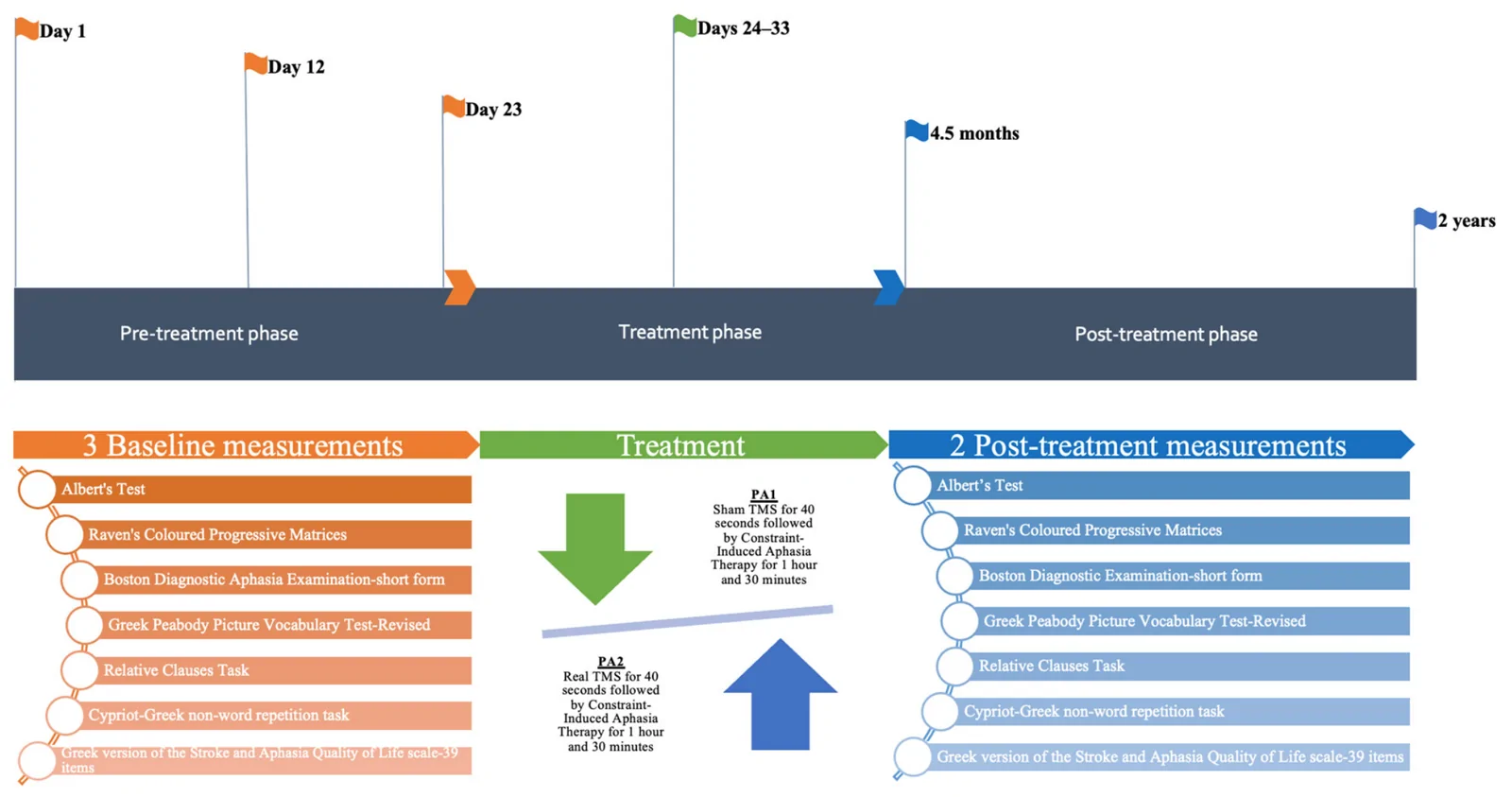
Experimental timeline of the study.

A research timeline was followed (see Figure 2) ensuring both participants underwent similar pre- and post-therapy procedures.

Each participant was assessed three times for baseline measures prior to the commencement of treatment, with 10-day intervals between evaluations, during which neither SALT nor rTMS was administered. Following baseline measurements, the participants underwent a 10-day treatment protocol over two consecutive weeks (excluding weekends). Each session began with either sham (PA1) or real (PA2) cTBS administered first—before mCIAT-GR—to leverage the priming window during which cTBS-induced cortical excitability changes are hypothesized to enhance the neural encoding of subsequent language practice, consistent with the sequencing recommendations supported by Zumbansen et al. [23]. The cTBS session had an allocated total time of 15 min. This was immediately followed by 1 h and 30 min of mCIAT, resulting in a total daily treatment duration of one hour and 45 min (see Figure 2). Post-treatment, two follow-up evaluations were conducted: the first at four and a half months post-treatment and the second at two years post-treatment. The rTMS protocol was administered by the first author (AMG). The mCIAT protocol was administered by the second author, an SLT clinician (PP) acting as the conversational partner, along with an assistant (therapist coach). Both were blind to the treatment type. Neither therapist had a prior therapeutic relationship with the participants before the study.

The assessment and treatment procedures took place at the Brain and Neurorehabilitation Lab at the Rehabilitation University Clinic of the Cyprus University of Technology (CUT) in Limassol, Cyprus. A certified SLT, blinded to the cTBS condition (real or sham), conducted the language and cognitive assessments at three baseline time periods and two follow-up periods. QoL was measured at the first baseline and second follow-up, but PA1 only provided the QoL questionnaire at the first baseline.

### 2.4. Treatment

#### 2.4.1. Continuous Theta Burst Stimulation

Surface electromyography (EMG) was utilized to determine the resting motor threshold (RMT) of PA2 by placing electrodes over the first dorsal interosseous (FDI) muscle of his left hand. The coil was positioned over the right primary motor cortex and stimulated with a single pulse at the optimal site to produce a motor evoked potential (MEP) of at least 50 µV in five out of ten repeated stimulations of the left hand’s FDI. The motor threshold levels, indicators of cortical excitability, were used to calculate stimulation parameters. PA2 underwent cTBS at 80% of their individual RMT using the Magstim Rapid2^®^ stimulator (Magstim Co., Wales, UK) connected to a 70 mm Double Air Film Coil. Stimulation parameters were set according to Wassermann’s recommendations [42]. A T1-weighted MRI scan of PA2 was taken prior to stimulation. A frameless stereotactic neuronavigation system (ANT NEURO, Magstim TMS system, The Magstim Company Ltd., Whitland, Carmarthenshire, UK) guided the coil position, utilizing the patient’s MRI to precisely target the stimulation area. Following Huang et al.’s [43] protocol, PA2 received inhibitory cTBS targeting the pTr of the right IFG (homologous BA45). Unlike conventional low-frequency rTMS (1 Hz), which achieves inhibitory effects through sustained repetitive stimulation trains, cTBS induces inhibitory modulation through brief bursts of high-frequency stimulation delivered at theta frequency. Specifically, cTBS consists of triplets of 50 Hz pulses delivered every 200 milliseconds, resulting in a 40 s stimulation train comprising 600 pulses in total. The reported 15 min TMS session duration included participant positioning, coil placement, and preparation procedures, whereas the active cTBS stimulation itself was completed within the 40 s train. PA2 received a total of ten daily stimulation treatments over two consecutive weeks, excluding weekends. For PA1, sham cTBS was administered for the same 40 s duration each time, with the coil centre positioned parallel to the vertex (tilted 90° away from the skull surface) so that the magnetic field did not penetrate the cortex, while the coil handle and positioning procedure remained identical to the real condition to maintain blinding. Both participants experienced the same total session duration (15 min).

#### 2.4.2. Modified Constraint-Induced Aphasia Therapy-Greek (mCIAT-GR)

The protocol was designed to be implemented with one patient and two SLTs, given the limited availability of participants, which often presents a challenge in clinical research especially for under-represented languages and clinical settings within countries with emerging aphasia research. The mCIAT-GR protocol was administered over two weeks, with therapy conducted five days per week, excluding weekends. The total intensity was seven and a half hours per week, with one and a half hours of therapy daily, resulting in a total dosage of 15 h. The treatment involved two SLTs: a conversational partner who sat opposite the participant and a therapist coach who sat next to the participant to aid. The therapy utilized 96 black-and-white cards, consisting of high- and low-frequency nouns associated with verbs. The treatment stimuli were developed by the first (AMG) and third (MK) authors for each treatment level, utilizing the Snodgrass & Vanderwart [44] picture set. These pictures were evaluated for psychometric properties such as name agreement, visual complexity, and age of acquisition by Dimitropoulou et al. [45]. Additionally, word frequency data was sourced from the GreekLex database by Ktori et al. [46]. There were 12 levels of treatment designed to progressively challenge the participants’ language abilities.

### 2.5. Analysis

Effect sizes were not calculated in the present study because conventional single-case indices, such as Standardized Mean Change (SMC), Non-overlap of All Pairs (NAP), Improvement Rate Difference (IRD), and standardized mean difference (Cohen’s d) [47], are most informative when based on a larger number of repeated observations across study phases. Although baseline performance was generally stable, the present feasibility pilot included only three baseline assessments, two participants, and several outcome measures demonstrating restricted score ranges or near-ceiling performance. Under these conditions, small changes in raw scores may disproportionately influence quantitative effect size estimates and potentially overstate treatment-related change. Consequently, visual analysis, complemented by Standard Error of Measurement (SEM) interpretation, was considered the most appropriate and conservative analytical approach for evaluating individual performance over time. Visual analysis offers a more nuanced and precise reflection of participants’ performance over time, allowing for clearer interpretation of both stable patterns and subtle changes. This approach ensured that small variations in the baseline phase did not distort the overall evaluation of the data. The SEM is reported for each measure as a practical index of measurement precision, allowing readers to judge whether observed score changes fall within or beyond expected measurement noise. This provides a more principled basis for interpreting stability claims than verbal description alone. Regarding Weighted Statistics (WEST; [48]), a method previously applied in related work from this research group [26,49], the authors acknowledge that dismissing this approach requires explicit justification rather than a brief citation. WEST is most informative when there is measurable variability in the data to weight and when the performance trajectory shows directional change. In the present study, the majority of measures across both participants show near-ceiling performance with minimal variance across all five timepoints, meaning the conditions under which WEST provides maximal discriminative power—a reasonably distributed score range and detectable improvement trend—are precisely the conditions absent here. Applying WEST to data in which scores cluster within 1–2 points of the ceiling across all timepoints would not reveal treatment effects that visual analysis cannot; it would instead produce effect estimates that are artefactually inflated by floor-level variance, exactly the concern that motivated the choice of visual analysis in the first place. Future adequately powered single-case or controlled studies with more extensive repeated measurements may benefit from complementary quantitative approaches, including effect size indices and reliable change analyses.

## 3. Results

### 3.1. Feasibility Outcomes

Both participants completed the full 10-day treatment protocol (100% session adherence). No adverse effects associated with cTBS or mCIAT were reported by either participant at any point during or after the treatment period. Both participants tolerated the stimulation and therapy sessions without complaint, and no safety concerns arose. The combined cTBS-plus-mCIAT-GR protocol was therefore deliverable, safe, and acceptable in a Cypriot clinical rehabilitation context, with one participant (PA1) receiving sham cTBS and one (PA2) receiving real cTBS, both in combination with mCIAT-GR. These feasibility findings are the primary positive outcome of the present pilot and provide the foundation for a future powered efficacy trial. Regarding QoL as a secondary feasibility-related outcome, PA1 did not return the SAQOL-39g at follow-up 2 as requested (discussed in the QoL section below), which is itself a feasibility signal regarding respondent burden and data-collection procedures to be addressed in future work.

### 3.2. Linguistic and Cognitive Measures

**Figure 3:**
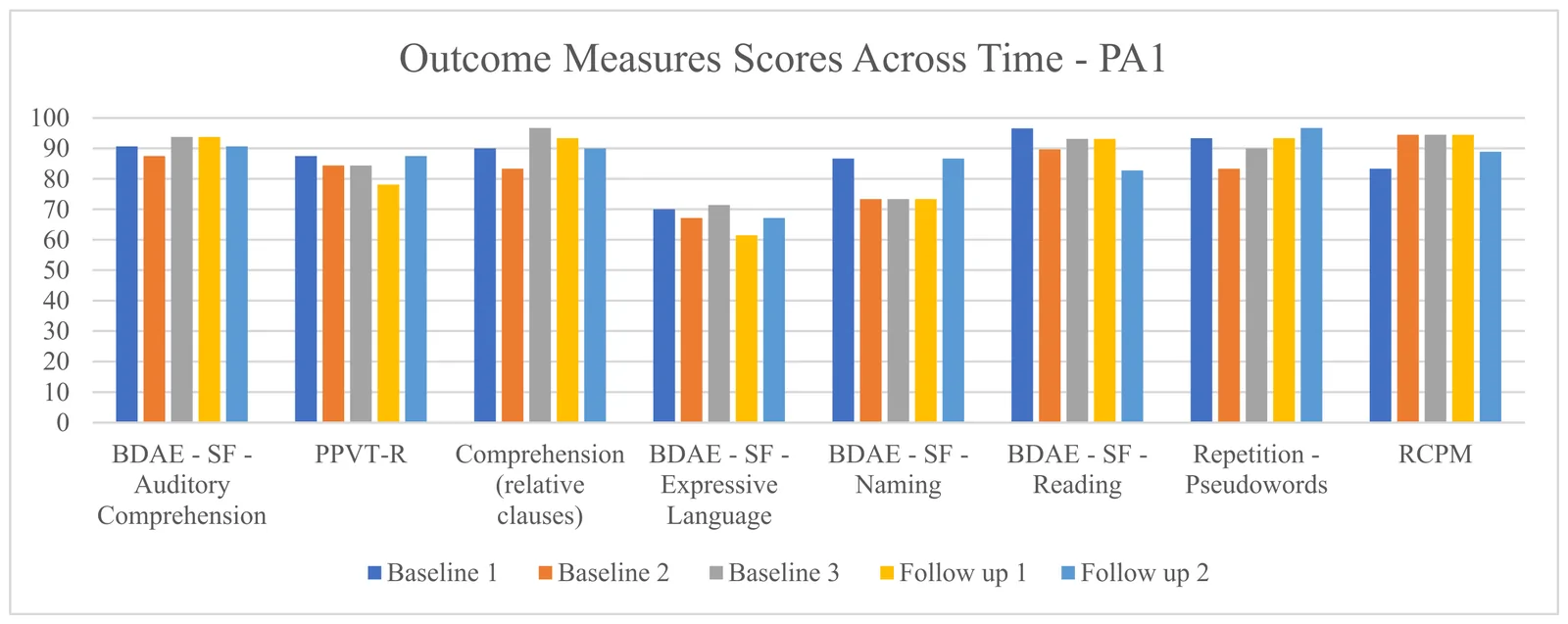
Linguistic and Cognitive Outcome Measures for PA1.

**Figure 4:**
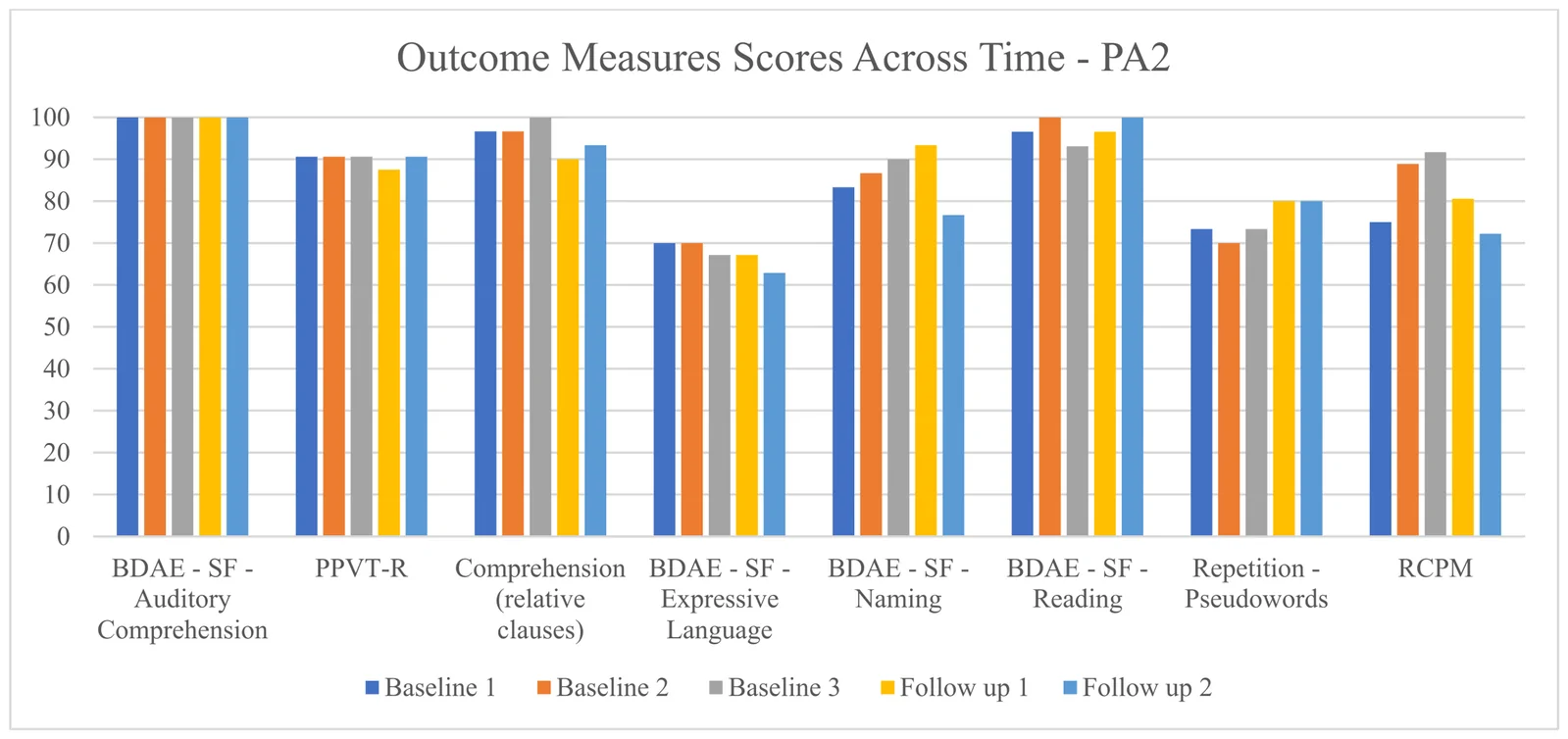
Linguistic and Cognitive Outcome Measures for PA2.

Overall, PA1 showed greater variability across assessment tasks, while PA2 maintained a more stable trajectory with slight fluctuations. With regard to auditory comprehension, PA2 remained stable across all time points, while PA1 exhibited minor fluctuations without lasting improvement post-intervention. Single-word comprehension in PA1 showed a decline at follow-up 1 before recovering at follow-up 2, indicating some variability, whereas PA2 remained largely stable with only a minor drop in scores at follow-up 1. Relative clause comprehension in PA1 showed minor fluctuations over time, while PA2 demonstrated stable performance with minor fluctuations. Expressive language in PA1 showed fluctuations, with a decline at follow-up 1 and partial recovery at follow-up 2, whereas PA2 remained more stable but showed a gradual decline over time. Naming abilities in PA1 fluctuated, whereas PA2 showed a notable pattern: naming improved to 14/15 at follow-up 1 before declining to 11.5/15 at follow-up 2, ending below the pre-treatment baseline—a trajectory that must be interpreted with caution given the two-year interval between follow-up 1 and follow-up 2, during which many uncontrolled factors could account for change. Reading performance indicated a potential decline for PA1 at follow-up 2, while PA2 remained consistently stable. Non-word repetition remained largely stable for both participants, with slight fluctuations. RCPM scores improved during baseline measurements; at follow-up 2, PA2’s RCPM score declined from 33 (Baseline 3) to 26, a 7-point drop in non-verbal cognition at the two-year mark. This decline should not be attributed to the intervention given the two-year interval; it is more plausibly attributable to ageing or unrelated cognitive changes over that period. Notably, the BDAE expressive language subscale was the one measure that was not at ceiling—both participants scored approximately 70% (24.5/35) at baseline—meaning the tool had sufficient sensitivity to detect change if any had occurred. Participant performance across all measures is presented in Figure 3 and Figure 4 and Table 3. Standard Error of Measurement (SEM) analysis was conducted to determine whether observed score changes at follow-up exceeded the threshold expected from measurement noise alone.

SEM was estimated as SD√(1 − r) using published test–retest reliability coefficients for each instrument (BDAE-SF subtests: *r* = 0.87–0.89; PPVT-R: *r* = 0.94; Relative Clauses Task: *r* = 0.85; Non-word Repetition: *r* = 0.80; RCPM: *r* = 0.87). For PA1, the PPVT-R decline at follow-up 1 (−2.33; SEM = 1.00), the BDAE Expressive Language decline at follow-up 1 (−2.83; SEM = 1.88), and the BDAE Reading decline at follow-up 2 (−3.00; SEM = 0.87) all exceeded one SEM, indicating declines rather than improvements. PA1’s naming at follow-up 2 (+1.33 above baseline mean; SEM = 1.08) marginally exceeded the SEM in a positive direction. For PA2, the RCPM decline at follow-up 2 (−4.67; SEM = 1.98), the BDAE Expressive Language decline at follow-up 2 (−2.17; SEM = 1.88), and the naming decline at follow-up 2 (−1.50; SEM = 1.08) all exceed one SEM at the two-year time point, likely reflecting the extended follow-up interval rather than an intervention-related effect. Improvements in non-word repetition for PA2 at both follow-up points (+1.17; SEM = 0.98) marginally exceeded the threshold in a positive direction. Taken together, SEM analysis indicates that no systematic positive language changes exceeding measurement error were observed for either participant, consistent with the findings of the visual analysis.

### 3.3. Stroke and Aphasia Quality of Life Scale-39 Item (SAQOL-39)

There were no post-treatment data for PA1 on the SAQOL-39 as he did not provide the questionnaire at the final session; retrieval was attempted but the questionnaire was not returned. This represents a feasibility finding in itself: the absence of follow-up QoL data from one participant indicates that respondent burden or logistical barriers to self-administered questionnaire completion may affect data completeness in future studies, and structured in-clinic administration should be considered. PA2 showed improvement across the physical, communication, and psychosocial domains of the SAQOL-39g at follow-up 2 compared to baseline. However, this finding must be interpreted with caution for two reasons. First, PA2’s baseline SAQOL-39g score was lower than PA1’s, meaning that PA2’s improvement may partly reflect regression to the mean rather than a treatment effect—participants who start lower on a measure tend to show apparent improvement on re-testing. Second, the two-year interval between treatment and follow-up 2 means that many uncontrolled life events, health changes, and environmental factors could account for any change in QoL, and it is not possible to attribute the improvement to the intervention. The QoL finding for PA2 is therefore best described as a finding warranting further investigation in a study with standardized follow-up intervals, rather than evidence of treatment efficacy. The responses are shown in Table 4.

## 4. Discussion

The present pilot study had one primary objective: to evaluate the feasibility, safety, and acceptability of implementing an mCIAT protocol with or without rTMS in a Cypriot clinical context, providing detailed observations and outcomes to inform the design of future adequately powered clinical trials. Additionally, the study aimed to report on an mCIAT programme adapted for the Greek language and implemented with one patient and two SLTs, taking into consideration the limited availability of participants, a common challenge in clinical research.

Individuals with chronic anomic aphasia were deliberately selected to minimize clinical heterogeneity and provide a stable clinical context for evaluating the feasibility, safety, and protocol deliverability of the combined intervention. As spontaneous recovery is expected to have largely plateaued in the chronic stage, the likelihood that observed changes were attributable to natural recovery rather than the intervention was reduced. Given the exploratory nature of this pilot study, establishing feasibility in a well-defined patient group was considered an appropriate first step before evaluating the protocol in larger, more heterogeneous populations, including individuals with non-fluent or subacute aphasia.

In the context of this feasibility pilot study, success was not defined solely by treatment completion or the absence of adverse events. Feasibility was considered across multiple domains, including safety, protocol deliverability, participant adherence, acceptability of the intervention, and practical considerations related to implementation within a Cypriot clinical context. Treatment fidelity was also considered through adherence to the predefined mCIAT and cTBS procedures. These feasibility indicators were prioritized over efficacy outcomes, as the primary aim of the study was to evaluate whether the combined protocol could be implemented successfully and inform the design of future adequately powered clinical trials.

The two participants did not report any adverse effects associated with the treatment. Despite the evidence-based intensive behavioural therapeutic approaches applied, which focus on promoting neuroplasticity to facilitate language recovery, the findings did not reveal improvements across standardized language measures, particularly for expressive language, over the study period for either participant. These findings should be interpreted within the context of several methodological considerations that were recognized during the design of this feasibility study, including potential ceiling effects due to near-ceiling baseline performance, the absence of a comprehensive linguistic assessment, and the limited treatment dosage. Nevertheless, the possibility that the intervention itself may have produced limited or no measurable effects cannot be excluded and should be considered when interpreting these findings.

Given the exploratory nature and small sample size of this pilot study, the results should therefore be interpreted cautiously, with future adequately powered studies required to determine the efficacy of the combined intervention. Beyond methodological considerations, several patient-related factors may have influenced individual treatment responses. Aphasia recovery is highly variable and can be shaped by factors such as age, chronicity, severity, lesion location and extent, and prior therapy [26]. First, the advanced age of both participants may have reduced the brain’s ability to induce neuroplastic changes or new language networks required for expressive language recovery. Indeed, even though the association between age and recovery varies between studies [50], it has been purported that individuals who experience aphasia at a young age tend to have better outcomes in comparison to people who experience aphasia at an elderly age [4]. Also, both participants were in the chronic phase of stroke recovery. Particularly, PA1 presented with a longer post-stroke duration (108 months), whereas PA2 presented with a shorter chronicity period (38 months). The chronicity of aphasia presents a significant challenge in aphasia rehabilitation. Even though it has been suggested that (i) commencing aphasia therapy in the early phase post-stroke (i.e., <1 month post-stroke) results in better outcomes in comparison to starting therapy in the chronic phase (i.e., >6 months post-stroke) (RELEASE, [51]) and (ii) people with chronic aphasia reach a recovery plateau within the first year post-stroke [52,53]; there is evidence that treatment often enhances language abilities even several years post-stroke [54,55]. Furthermore, participants had mild anomic aphasia. While there is strong evidence supporting that higher severity is linked to lower rates of spontaneous recovery and poorer response to treatment [56], the mild severity of aphasia in the two participants in this case, provided limited room for improvement in expressive language making it harder to observe any significant changes after CIAT. Mild anomia was determined using the BDAE-SF [38], which is validated in Greek. However, this short form includes only a limited set of words, which may restrict its ability to fully capture the participants’ word-finding difficulties. A more comprehensive linguistic assessment could have potentially uncovered additional deficits, offering a clearer and more detailed understanding of their language profiles. This limitation also applies to the participants’ near-ceiling language performance, which might reflect both their actual language abilities and the reduced sensitivity of the assessment tools used. Nevertheless, all tools used in this study were validated in Greek and selected to capture a holistic linguistic profile of each participant. The Raven’s Coloured Progressive Matrices (RCPM), administered at baseline and repeated as a control variable for each participant, was used to account for fluctuations in non-linguistic cognitive functions. By incorporating the RCPM as a control variable, the analysis ensured that any observed changes in language performance were not confounded by general cognitive changes. This allowed for a more accurate interpretation of the linguistic results. It was hypothesized that if the RCPM remained stable across participants while improvements were observed in the linguistic domains, this would suggest that (i) CIAT, with or without TMS, enhanced language-specific gains, and (ii) the influence of placebo or training effects on the results was minimized, consistent with findings from prior research [26]. Regarding other parameters, lesion location and extent undoubtedly play a significant role in aphasia recovery. These factors influence the brain’s capacity for reorganization and response to treatment, potentially affecting the degree of improvement observed in each participant. Although it is reasonable to expect larger lesions to disrupt key language network hubs, and therefore have a greater negative effect on language recovery, the findings on lesion size remain contentious. While some studies indicate that larger lesions are linked to poorer outcomes in recovery [57], counter evidence claims that this is not always the case [58]. Damage to critical language areas in the brain, such as the frontoparietal and temporal lobes, as seen in PA1, can severely impair the neural networks necessary for language production. In comparison, PA2 had less extensive damage, yet improvements in expressive language were also absent, indicating that even mild damage may limit recovery in chronic aphasia. Furthermore, differences in previous SLT exposure and living circumstances may have influenced participants’ communication experiences, language use, and potential responsiveness to further intervention. PA1 had received substantial SLT input prior to enrolment, including 12 months of therapy three times weekly followed by an additional 24 months of therapy twice weekly, with SLT discontinued six years before participation in the present study. In contrast, PA2 had received a shorter period of SLT (three months, twice weekly), with therapy discontinued three years before enrolment. In addition, PA1 lived with his wife and family members, providing regular communication opportunities, whereas PA2 lived alone without a regular communication partner. These differences in rehabilitation history and everyday communication environment may have contributed to variability in language maintenance, functional communication demands, and individual recovery trajectories. However, given the small sample size, their specific contribution to treatment outcomes cannot be determined and should be considered when interpreting the findings. Last but not least, differences in participants’ everyday communication environments represent an additional factor that may have influenced outcomes. PA1 had regular communication opportunities with family members, whereas PA2 lived alone without a regular communication partner. Given that CILT relies on intensive verbal interaction and the generalization of trained communication behaviours beyond the therapy setting, differences in environmental language support may have affected functional language use and maintenance of gains. However, because this was a feasibility study with only one participant in each stimulation condition, the influence of communication environment cannot be separated from the effects of the intervention itself. Future efficacy trials should consider assessing and controlling for communication partner availability and real-world language exposure. Overall, despite many studies motivated to identify factors that predict treatment outcomes in aphasia, the current evidence still does not enable clinicians and researchers to customize treatments or reliably predict language improvements from initial assessments [59].

Crucially, the incorporation of a QoL measure in this study produced the most notable finding: PA2 showed improvement across all SAQOL-39g domains at Follow-up 2. The dissociation between stable standardized language scores and apparent QoL improvement is theoretically interesting: it is consistent with the hypothesis that mCIAT, by embedding language practice within a communicative social interaction, may improve perceived functional communication and psychosocial well-being even when formal language test scores do not change. This would align with the distinction between impairment-level and activity/participation-level outcomes in aphasia rehabilitation [60]. Overall, the interpretation of the two-year follow-up outcomes requires particular caution. Both participants underwent follow-up linguistic assessments at approximately four and a half months, and two years after the intervention; however, only PA2 completed the SAQOL-39g questionnaire at the two-year assessment. The extended interval between treatment completion and final reassessment introduces uncertainty, as numerous uncontrolled clinical and contextual factors unrelated to the intervention may have influenced language performance, cognitive outcomes, and QoL over this period. Therefore, the participants’ two-year findings, including QoL outcomes, should be considered exploratory observations of long-term status rather than direct indicators of treatment efficacy. Nevertheless, these data provide preliminary information regarding the feasibility and potential value of long-term follow-up after intensive aphasia rehabilitation. Future studies should incorporate predefined and comparable follow-up intervals, systematic monitoring of relevant factors, and appropriate control conditions to enable more robust evaluation of long-term outcomes.

The adaptation of mCIAT for Greek-speaking individuals represents an important contribution of the present study; however, further validation is required before mCIAT-GR can be considered a fully transferable clinical resource. The adaptation process considered the linguistic characteristics of Greek and the need for culturally and clinically appropriate treatment stimuli. In our protocol we incorporated modifiers (specifically adjectives), and verbs in both the active and passive tense aiming for a comprehensive language-specific approach that addresses various linguistic aspects relevant also to aphasia language therapy practices. Moreover, the protocol was structured to accommodate single-patient sessions, recognizing the practical challenges of scheduling multiple individuals with similar aphasia profiles simultaneously. In this approach, as in any CIAT programme, receptive language tasks were integral to the development of the protocol. By integrating these receptive components into the CIAT regimen, the principle that targeting both expressive and receptive language leads to better overall outcomes was adhered to. This approach is supported by research evidence, such as the findings from Brady et al. [60], highlighting that mixed expressive-receptive therapies are associated with the greatest language improvements. The one-patient, two-therapist format adopted in the present study represents a meaningful methodological adaptation from the original group CIAT model that deserves explicit discussion. Standard CIAT is delivered in groups of three or more patients, which serves multiple functions: it creates a genuine communicative need to request information from a peer, it provides social modelling of constrained verbal production, and it distributes the pragmatic demands of the task across participants. The dual-therapist single-patient format used here—in which one therapist acted as a conversational partner and a second served as a therapist coach—was necessitated by the difficulty of recruiting multiple participants with closely matched aphasia profiles in the Cypriot clinical context. This recruitment challenge is itself a feasibility finding. In terms of protocol fidelity, the constraint principle was maintained: the participant was required to produce verbal utterances to receive cards, and compensatory strategies were discouraged. However, the pragmatic demands of the task differ from the group format in important ways: the social pressure of communicating with a peer is not replicated when communicating with a known therapist, and the patient cannot observe peer modelling of constrained verbal production. It is also possible that the SLT’s cueing role—providing semantic and phonological cues when the participant struggled—reduced the communicative pressure that is central to the constraint mechanism. Future studies should report the frequency and type of cues provided as a treatment fidelity measure and should explore whether the dual-therapist format affects outcomes relative to the group format. A detailed description of the mCIAT-GR protocol, including treatment materials and implementation procedures, is currently being prepared for formal publication. The present study provides preliminary feasibility evidence for its clinical implementation, while the forthcoming protocol publication will support replication and wider clinical use.

As for the neuromodulation component of the present study, rTMS has the potential to increase cortical plasticity and improve language outcomes in patients with post-stroke aphasia both as an adjunct therapy and as a standalone treatment (see [61] and references within). The Third Stroke Recovery and Rehabilitation Roundtable (SRRR3) [62] aiming to address barriers to translating research on non-invasive brain stimulation, like TMS into clinical practice, found that for aphasia many trials have applied non-standardized measures with unclear psychometric properties, limiting the utility and generalizability of outcomes. As a result, research on TMS and aphasia is not currently sufficiently reliable to influence rehabilitation practice. In our study, as both participants received mCIAT and differed only in the type of cTBS administered (real versus sham), the present design does not allow the independent contribution of cTBS to be fully disentangled from the effects of mCIAT. However, this approach was consistent with the primary objective of this feasibility pilot study, which was to determine whether a combined cTBS-plus-mCIAT protocol could be delivered safely and acceptably in a Cypriot clinical context, rather than to establish the individual efficacy of each treatment component. Future adequately powered randomized controlled studies incorporating appropriate comparison conditions (e.g., mCIAT alone, sham cTBS plus mCIAT, and active cTBS plus mCIAT) will be necessary to determine the specific contribution of neuromodulation and any potential synergistic effects between treatment components.

The 10-day protocol provided in the current study may not have been of sufficient intensity or duration to achieve measurable improvements in expressive language. Aligning with evidence-based guidelines for post-stroke aphasia recovery and with regard to intensity (hours/week), dosage (total hours), frequency (days/week), and duration (weeks) [51], it is promising that optimal language recovery may occur with frequent, intensive therapy of 2–4 h per day, or 9+ hours per week, with a total treatment dosage ranging from 20 to 50 h. Overall, with regard to the treatment protocol, the following adjustments are recommended to more closely align with evidence-based guidelines aimed at optimizing language recovery, to better assess the potential effectiveness of the treatment in enhancing therapeutic outcomes:

- Treatment duration (mCIAT+cTBS/iTBS): 3 weeks.
- Frequency: 5 days per week (no sessions on weekends).
- Combined Treatment Intensity:
  ○ cTBS first followed by mCIAT: 40 s of cTBS daily, followed by 3 h of mCIAT with a 5 min break after each hour of therapy;
  ○ iTBS first followed by mCIAT: 200 s of iTBS daily, followed by 3 h of mCIAT with a 5 min break after each hour of therapy.
- Treatment Dosage:
  ○ cTBS + mCIAT: 45.17 h total over 3 weeks;
  ○ iTBS + mCIAT: 45.83 h total over 3 weeks.

As with any study, the present study has some limitations. The Greek BDAE-SF is a screening tool, and both participants entered the study with mild anomic aphasia and near-ceiling performance on many subtests; this mismatch between tool sensitivity and participant profile is acknowledged as a primary design limitation. Future studies with similar participants should prioritize the full BNT, discourse productivity measures (e.g., correct information units, main concept analysis from connected speech samples), and morphosyntactic production tasks appropriate for Greek. Also, the SCRIBE guidelines for single-case reporting in behavioural interventions [63] recommend a minimum of five baseline data points to establish stability. The three baseline assessments used in the present study fall below this recommendation and therefore represent a design limitation. However, this decision was influenced by feasibility considerations, including the potential risk of participant dropout, particularly for PA2, who lived alone, approximately two hours away from the study setting, and faced considerable travel-related costs. Also, modern neuroimaging methods (e.g., fMRI or EEG), are valuable for tracking neuroplastic changes, but since these were not employed one cannot speculate on any neuroplastic changes that may have occurred. Crucially, without neuroimaging data it is not possible to make any claims about whether cTBS to the right IFG produced the intended inhibitory effects on right-hemisphere language processing, whether hemispheric lateralisation of language shifted over the treatment period, or whether any neural reorganization occurred. The interhemispheric competition account—whereby cTBS suppression of right IFG disinhibits left-hemisphere perilesional networks—is a hypothesis grounded in prior neuroimaging studies [31,33], not a finding of the present study. Overall, given the feasibility nature of this pilot study and the small sample size, including only one participant in each stimulation condition, comparisons between real and sham stimulation conditions should be interpreted with caution. Future adequately powered randomized controlled trials are required to investigate the comparative efficacy of real versus sham stimulation. Last but not least, future efficacy trials should incorporate more rigorous procedures to evaluate the success of participant blinding. Specifically, the use of dedicated active sham coils that closely mimic the somatic sensations of active stimulation without inducing cortical modulation, together with validated blinding credibility questionnaires, would provide a more robust assessment of blinding integrity and reduce the risk of participant unblinding.

The findings of the present feasibility study support the following specific design for a future powered efficacy trial. Target participants should be native speakers of Greek, be right-handed, and have no prior CIAT exposure; a target sample of *n* = 10–12 per arm is suggested based on effect sizes from comparable published feasibility-to-efficacy transitions in the rTMS-plus-SLT literature [17,29]. The assessment battery should include the full Boston Naming Test (BNT), connected speech productivity measures (correct information units; main concept analysis), the Relative Clauses Task and a morphosyntactic production battery appropriate for Greek, the full BDAE (not the short form), and the SAQOL-39g administered in-clinic at standardized intervals (e.g., baseline, immediately post-treatment, 3-month follow-up, 6-month follow-up and 1-year follow-up). Treatment dosage should align with RELEASE [51] guidelines: a minimum of 3 h per day for 3 weeks (around 45 h total), with rTMS administered before each mCIAT session. A neuroimaging component—pre- and post-treatment task-based fMRI measuring language lateralisation and perilesional activation—would allow the hypothesized neurophysiological mechanism to be tested directly. Blinding should be formally assessed using a validated credibility scale. A SCRIBE-compliant single-case series or a randomized crossover design with matched sham and real cTBS arms would both be appropriate designs for this population size.

## 5. Conclusions

In conclusion, while participants in this pilot feasibility study did not show improvements in expressive language abilities following mCIAT or its combination with cTBS, the protocol was delivered safely and with full adherence across both participants—establishing the primary feasibility objectives of the study. Several factors may have contributed to the absence of observable language improvements, including ceiling effects, limited assessment sensitivity, insufficient treatment dosage, and the possibility of minimal intervention effects. Therefore, the efficacy of the combined intervention remains to be established in future larger-scale studies. Adjustments in the duration, frequency, intensity, and dosage of the combined protocol, as specified in the future directions section above, are necessary before efficacy can be meaningfully evaluated. While standardized language tests did not capture improvements, the QoL finding for PA2—interpreted cautiously given the two-year follow-up interval and regression-to-mean concerns—points to the importance of including activity/participation-level outcomes alongside impairment measures in future trials. Finally, this is the first study to report the results of a mCIAT program for Greek, a language with complex morphology. The authors hope this paves the way for future aphasia language treatment research in other underrepresented languages.

## Figures and Tables

**Table 1:** Aphasia studies combining rTMS with CIAT.

Study	Language/ Country	Participants	CIAT	TMS	Findings
[10]	English/USA	13 with chronic aphasia post- ischemic stroke (8 Anomic, 2 Broca, 1 Global/Conduction/ Wernicke’s each)	45–60 min of mCIAT over 10 consecutive weekdays	Neuronavigated iTBS for 200 s. at 80% of AMT using a figure of 8 coil prior to CIAT	Improvements in naming and apraxia of speech, and in perception of everyday communicative ability
[17]	Finnish	17 participants with chronic aphasia (8 Anomic, 5 Conduction, 3 Broca, 1 Transcortical Motor)	ILAT 3 h daily over 10 days (not on weekends)	Neuronavigated 1 Hz rTMS for 20 min. at 90% of the MT using a figure of 8 cooled coil; or sham prior to ILAT	ILAT associated with significant improvement on expressive language abilities (WAB/BNT/ANT) but not rTMS
[29]	English/USA	12 participants with chronic aphasia (7 Anomic, 2 Broca; 1 Conduction, 1 Global)	45 min of mCIAT over 10 consecutive weekdays	iTBS for 200 s. at 80% of AMT using a figure of 8 coil prior to CIAT; fMRI-identified residual left hemispheric language area for each participant	Improvements in naming (BNT) and WAB scores
[34]	English/USA	2 participants with chronic nonfluent aphasia	3 h of mCILT over 10 consecutive days	1 Hz rTMS was applied for 20 min. using an air-cooled, figure 8-shaped TMS coil prior to mCILT	Significant improvement in naming pictures of objects or actions, and in propositional speech (total number of narrative words and different nouns) at 1–2 months post-TMS+mCILT for each patient.

Key: AMT: active motor threshold; ANT: action naming test; BNT: Boston naming test; CIAT: constraint-induced aphasia therapy; CILT: constraint-induced language therapy; ILAT: intensive language action therapy; iTBS: intermittent theta burst stimulation; mCIAT: modified CIAT; mCILT: modified CILT; MT: motor threshold; rTMS: repetitive transcranial magnetic stimulation; WAB: Western Aphasia Battery.

**Table 2:** Demographic and clinical characteristics of the participants.

Participant	Sex	Age (Years)	Handedness	Education (Years)	Type of Stroke	Months Post Stroke	Lesion Site (Left Hemisphere)	Type and Severity of Aphasia	Hemiplegia	SLT Prior to Enrolment	Termination of SLT	Living Situation	Communication Partner
1	M	57	right	18	ischemic	108	- Extensive brain parenchymal loss involving the left frontoparietal lobe extending inferiorly at the anterior left temporal lobe - Mild perifocal brain gliosis - Mild ex vacuo dilatation mainly of the frontal horn and body of the left lateral ventricle - Asymmetry of the cerebral peduncles noted, with the left side significantly smaller than the right, also involving the pons-compatible with a left-sided Wallerian degeneration	mild anomic	absence	12 months: 3 times per week 24 months: 2 times per week	6 years before enrolment	lives with his wife	child, and son-in-law
2	M	58	right	12	ischemic	38	- A small area of brain parenchymal loss involving the left parietal lobe with mild perifocal gliosis	mild anomic	right	3 months: 2 times per week	3 years before enrolment	lives alone	none

**Table 3:** Raw Scores Comparison of Language and Cognitive Outcomes in PA1 and PA2 Across Timepoints.

Outcome Measures		Baseline 1	Baseline 2	Baseline 3	Follow Up 1	Follow Up 2
BDAE-SF-Auditory Comprehension	PA1	29/32	28/32	30/32	30/32	29/32
	PA2	32/32	32/32	32/32	32/32	32/32
PPVT-R	PA1	28/32	27/32	27/32	25/32	28/32
	PA2	29/32	29/32	29/32	28/32	29/32
Comprehension (relative clauses)	PA1	27/30	25/30	29/30	28/30	27/30
	PA2	29/30	29/30	30/30	27/30	28/30
BDAE-SF-Expressive Language	PA1	24.5/35	23.5/35	25/35	21.5/35	23.5/35
	PA2	24.5/35	24.5/35	23.5/35	23.5/35	22/35
BDAE-SF-Naming	PA1	13/15	11/15	11/15	11/15	13/15
	PA2	12.5/15	13/15	13.5/15	14/15	11.5/15
BDAE-SF-Reading	PA1	28/29	26/29	27/29	27/29	24/29
	PA2	28/29	29/29	27/29	28/29	29/29
Repetition-Pseudowords	PA1	14 /15	12.5/15	13.5/15	14/15	14.5/15
	PA2	11/15	10.5/15	11/15	12/15	12/15
RCPM	PA1	30/36	34/36	34/36	34/36	32/36
	PA2	27/36	32/36	33/36	29/36	26/36

Key: BDAE-SF: Boston Diagnostic Aphasia Examination-Short Form; PA: Participant; PPVT-R: Peabody Vocabulary Test-Revised; RCPM: Raven’s Coloured Progressive Matrices.

**Table 4:** Stroke and Aphasia Quality of Life scale-39 item outcomes at follow-up compared to baseline.

Quality of Life	Participant 1		Participant 2	
	Pre TMS	Follow Up 2	Pre TMS	Follow Up 2
SAQOL-39g Mean score	3.23	—	2.85	4.02
Physical score	3.38	—	2.69	3.5
Communication score	2.86	—	2.29	4
Psychosocial score	3.25	—	3.25	4.56

Note: Missing data are indicated by an em dash (—). For PA1, the SAQOL-39g follow-up assessment was not available because the participant did not return the questionnaire.

## Data Availability

The data presented in this study are available on reasonable request from the corresponding author.
